# Draft Genome Sequence of the Terrestrial Cyanobacterium Scytonema millei VB511283, Isolated from Eastern India

**DOI:** 10.1128/genomeA.00009-15

**Published:** 2015-03-05

**Authors:** Diya Sen, Mathu Malar Chandrababunaidu, Deeksha Singh, Neha Sanghi, Arpita Ghorai, Gyan Prakash Mishra, Madhavi Madduluri, Siba Prasad Adhikary, Sucheta Tripathy

**Affiliations:** a Structural Biology and Bioinformatics Division, Council of Scientific and Industrial Research, Indian Institute of Chemical Biology, Kolkata, West Bengal, India; b Fakir Mohan University, Vyasa Vihar, Nuapadhi, Balasore, Odisha, India

## Abstract

We report here the draft genome sequence of Scytonema millei VB511283, a cyanobacterium isolated from biofilms on the exterior of stone monuments in Santiniketan, eastern India. The draft genome is 11,627,246 bp long (11.63 Mb), with 118 scaffolds. About 9,011 protein-coding genes, 117 tRNAs, and 12 rRNAs are predicted from this assembly.

## GENOME ANNOUNCEMENT

Cyanobacteria are photosynthetic Gram-negative bacteria that are found in marine water and freshwater, as well as under diverse terrestrial habitats. They can naturally produce a wide range of compounds ranging from pigments, antibiotics, and food supplements to biofuels and biodegradable plastics (1). Current research is aimed at genetically increasing the yields of biofuels, such as ethylene, alkane, 1-butanol (2), and fatty alcohols (3), so as to make them economically more feasible. Scytoscalarol is an antimicrobial terpene containing several different cyclic peptides functioning as protease inhibitors isolated from Scytonema sp. strain UTEX 1163 (4, 5). Another strain of Scytonema has been shown to produce an extracellular sheath pigment called scytonemin that absorbs UV radiation and allows the organism to survive in high-energy solar radiation (6). This compound can be used in the production of marketable sunscreen products (7). Despite having useful applications in drug and cosmetic development, Scytonema is a genus that is unexplored for its bioprospecting potential.

*Syctonema millei* VB511283, a terrestrial cyanobacterium, was isolated from biofilms growing on the exterior of stone monuments in Santiniketan, eastern India (8). It was maintained in BG11 medium at room temperature (approximately 26°C) in a cycle of 16 h of light/8 h of darkness, without shaking. The culture formed dark green mats on solid slants but was ribbon-like in liquid medium.

Genomic DNA was isolated with the UniFlex bacterial DNA isolation kit (Genei, USA). A total of 404 ng of DNA was used for sequencing. Sequencing was carried out on an Illumina HiSeq platform (Genotypic Technologies, India) using paired-end and mate-pair libraries. The paired-end library consisted of an approximately 300-bp insert, with a read length of 151 bp and coverage of 291×, resulting in 44 million reads. The mate-pair library consisted of an approximately 3,000-bp insert, with a read length of 101 bp and 20× coverage, yielding 4.4 million reads. The raw reads were cleaned by SGA and TagDust (9). Genome assembly was carried out on cleaned reads from both libraries using AllPaths-LG (10). This resulted in 118 scaffolds, with an *N*_50_ of 2,155,310. The largest scaffold was 3,887,589 bp long, and the smallest was 3,012 bp long; the draft genome is 11,627,246 bp and has a G+C content of 51%.

Genome annotation was performed with the Prokaryotic Genome Annotation Pipeline (PGAP) at NCBI (http://www.ncbi.nlm.nih.gov/genome/annotation_prok/). There are 9,011 protein-coding genes, 1,300 pseudogenes, 2 clustered regularly interspaced short palindromic repeat (CRISPR) arrays, 117 tRNAs, 12 rRNAs, and 2 noncoding RNAs (ncRNAs). tBLASTn carried out against cyanobacterial core orthologs (11) revealed the presence of 382 of the 384 core orthologs. Besides the core orthologs, the genome of S. millei encodes several types of multidrug resistance systems, such as macrolide/bacitracin/multidrug ABC transporters, the acriflavin resistance gene (*acrB*), and β-lactamases. A polyketide synthase gene bearing 66% identity at the amino acid level to its homolog in Nostoc punctiforme PCC 73102 (accession no. WP_012409829.1) was identified. This strain can thus be explored for the production of pharmacologically important polyketides.

### Nucleotide sequence accession number.

The whole-genome sequence and annotation data for S. millei VB511283 have been submitted to GenBank under the accession no. JTJC00000000.

## References

[1] Abed RMM, Dobretsov S, Sudesh K 2009 Applications of cyanobacteria in biotechnology. J Appl Microbiol 106:1–12. doi:10.1111/j.1365-2672.2008.03918.x. 19191979

[2] Ducat DC, Way JC, Silver PA 2011 Engineering cyanobacteria to generate high-value products. Trends Biotechnol 29:95–103. 21211860 10.1016/j.tibtech.2010.12.003

[3] Ruffing AM 2014 Improved free fatty acid production in cyanobacteria with *Synechococcus* sp. PCC 7002 as host. Front Bioeng Biotechnol 2:17. doi:10.3389/fbioe.2014.00017. 25152890 PMC4126656

[4] Mo S, Krunic A, Pegan SD, Franzblau SG, Orjala J 2009 An antimicrobial guanidine-bearing sesterterpene from the cultured cyanobacterium *Scytonema* sp. J Nat Prod 72:2043–2045. doi:10.1021/np900288x. 19888742 PMC2988765

[5] Krunic A, Vallat A, Mo S, Lantvit DD, Swanson SM, Orjala J 2010 Scytonemides A and B, cyclic peptides with 20S proteasome inhibitory activity from the cultured cyanobacterium *Scytonema hofmanii*. J Nat Prod 73:1927–1932. doi:10.1021/np100600z. 21058727 PMC3074188

[6] Dillon JG, Castenholz RW 1999 Scytonemin, a cyanobacterial sheath pigment, protects against UVC radiation: implications for early photosynthetic life. J Phycol 35:673–681. doi:10.1046/j.1529-8817.1999.3540673.x.

[7] Proteau PJ, Gerwick WH, Garcia-Pichel F, Castenholz RW 1993 The structure of scytonemin, an ultraviolet sunscreen pigment from the sheaths of cyanobacteria. Experientia 49:825–829. doi:10.1007/BF01923559. 8405307

[8] Keshari N, Adhikary SP 2013 Characterization of cyanobacteria isolated from biofilms on stone monuments at Santiniketan, India. Biofouling 29:525–536. doi:10.1080/08927014.2013.794224. 23679119

[9] Coil D, Jospin G, Darling AE 2014 A5-miseq: an updated pipeline to assemble microbial genomes from Illumina MiSeq data. Bioinformatics, in press. doi:10.1093/bioinformatics/btu661. 25338718

[10] Butler J, MacCallum I, Kleber M, Shlyakhter IA, Belmonte MK, Lander ES, Nusbaum C, Jaffe DB 2008 ALLPATHS: *de novo* assembly of whole-genome shotgun microreads. Genome Res 18:810–820. doi:10.1101/gr.7337908. 18340039 PMC2336810

[11] Larsson J, Nylander JA, Bergman B 2011 Genome fluctuations in cyanobacteria reflect evolutionary, developmental and adaptive traits. BMC Evol Biol 11:187. doi:10.1186/1471-2148-11-187. 21718514 PMC3141437

